# Comparison of Prophylactic Phenylephrine Infusion and Rescue Bolus Administration for Maintaining Blood Pressure During Spinal Anaesthesia for Caesarean Delivery in Obese Parturient: A Randomised Control Trial

**DOI:** 10.21315/mjms2024.31.3.8

**Published:** 2024-06-27

**Authors:** Shamsul Kamalrujan Hassan, Pit Cui Wong, Praveena Seevaunnamtum, Sanihah Che Omar, Nik Abdullah Nik Mohamad

**Affiliations:** Department of Anaesthesiology and Intensive Care, School of Medical Sciences, Universiti Sains Malaysia, Kelantan, Malaysia

**Keywords:** obese, phenylephrine, Caesarean section, spinal anaesthesia, hypotension

## Abstract

**Background:**

Phenylephrine (PE) is one of the vasopressor used to treat hypotension during anaesthesia. The primary aim of this study was to compare the effect of prophylactic infusion and rescue bolus of PE on the haemodynamic changes during spinal anaesthesia (SA) for Caesarean section (CS) in obese parturients.

**Methods:**

A total of 74 obese parturients scheduled for elective CS under SA were randomised into two groups; Group A (*n* = 37) received prophylactic PE infusion starting at 50 μg min^−1^ and adjusted according to the given algorithm and Group B (*n* = 37) received 100 μg PE bolus to treat hypotension. The measured parameters were systolic blood pressure (SBP), diastolic blood pressure (DBP), mean arterial pressure (MAP), the total requirement of PE and neonatal Apgar score.

**Results:**

Six patients were excluded from the analysis due to missing data and only 68 were analysed. Group A showed significantly higher SBP, DBP and MAP than Group B (*P* < 0.05). The requirement of PE was higher in Group A than Group B [817.7 (265.7) μg versus 360.6 (156.0) μg; *P* = < 0.05]. Both groups had no difference in terms of the neonatal Apgar score.

**Conclusion:**

Prophylactic PE infusion provided better haemodynamic control than therapeutic boluses in obese parturients undergoing CS under SA.

## Introduction

Obesity and Caesarean section (CS) have been identified as independent risk factors for maternal morbidity and mortality (1). World Population Review 2019 has reported that Malaysia has the highest prevalence of obesity among adults in South-East Asia. About half (50.1%) of adult’s population in Malaysia who either overweight or obese. In other words, the number of obese parturients that require anaesthesia care is also at a rising trend.

Spinal anaesthesia (SA) should ideally be used for all CS unless contraindicated as it avoids the risks of maternal aspiration and difficult airway associated with general anaesthesia. However, sympathetic block due to SA can cause significant maternal hypotension. In addition, the fall in blood pressure (BP) is more severe among obese parturients than others. Therefore, vasopressor is required more frequently and in higher doses in these obese mothers (2, 3).

Phenylephrine (PE), an α-adrenoreceptor agonist, has become the vasopressor of choice for preventing and treating spinal anaesthesia-associated hypotension. The American Society of Anesthesiologists Practice Guidelines for Obstetric Anaesthesia published in 2016 recommends that PE should be considered in uncomplicated pregnancies without maternal bradycardia as it improves foetal acid-base status compared with the ephedrine (4, 5).

In 2018, The Association of Anaesthetists of Great Britain and Ireland (AAGBI) consensus statement published by Kinsella et al. (6) recommended a variable rate prophylactic infusion of PE should be started immediately after the intrathecal injection. A study conducted by Choudhary and Bajaj (7) found that prophylactic PE infusion leads to significantly better control of post-SA hypotension during CS. The study also shows that 1-min and 5-min neonatal Apgar scores between the two groups are statistically insignificant. A study by Shafeinia et al. (8) showed that there was less decrease in reduction of the mean arterial pressure (MAP), systolic blood pressure (SBP) and diastolic blood pressure (DBP) in the intervention group with 35 mcg/min of PE infusion than in the control group at different time points.

Despite current evidence supporting the use of PE infusion to prevent or treat post-SA hypotension, most previous research regarding PE infusion excluded the obese population. This is because obesity has profound effects on the mother’s physiology, which may alter the cardiovascular response to both SA and vasopressor therapy. In addition, maternal BMI may alter the pharmacodynamics effects of PE dosing when administered as a bolus or as a continuous infusion.

Therefore, the study aimed to compare the effect of prophylactic infusion and rescue bolus of PE on the haemodynamic changes and the requirement of PE during SA for CS in obese parturients population.

## Methods

This was a balanced randomisation (ratio1:1), a double-blind, parallel-group study conducted in Hospital Universiti Sains Malaysia (USM). After receiving approval from the Ethics Committee of USM and written consent, 74 parturients undergoing elective CS were recruited. Patients aged more than 18 years old and BMI between 30 kg/m^2^ and 40 kg/m^2^ were recruited in the study. The exclusion criteria were allergy to PE, hypertensive diseases of pregnancy (gestational hypertension and pre-eclampsia), chronic hypertension on anti-hypertensive treatment, cardiac disease, gestational age of less than 36 weeks and height less than 140 cm or more than 180 cm.

Consented parturient were randomised into two groups using computerised software randomisation. Group A received prophylactic PE infusion starting at 50 μg min^−1^ immediately after SA and Group B received bolus of 100 μg PE to treat the established hypotension. An opaque envelope contained the labelled A or B was opened by an assisted staff nurse who was not involved in the study. Then, the staff nurse prepared two blinded syringes per patient which were labelled as ‘Syringe set A’ and ‘Syringe set B’ according to the given envelope. The ensure blinding the possible combination that each parturient receives are as follows: Syringe set A was a pair of 60 mL syringe with PE 100 μg/mL for the infusion and a separate 10 mL syringe normal saline (NS) for bolus administration. Syringe set B was a pair of 60 mL syringe containing NS for the infusion with 10 mL syringe containing PE 100 μg/mL for bolus administration.

In the operation room, monitoring was applied, including non-invasive BP (NIBP), pulse oximetry (spO_2_) and electrocardiography (ECG). Their baseline BP, heart rate (HR) and spO_2_ were recorded. All patients were given standard SA using institutionally approved height-based dose of intrathecal heavy bupivacaine 0.5% in combination with intrathecal fentanyl 15 μg. If the height of the parturient was 140 cm–149 cm, an intrathecal dose of 8.5 mg of heavy bupivacaine 0.5% was provided. Also, if the parturient height was 150 cm–160 cm, the intrathecal dose 10 mg of 0.5% heavy bupivacaine was given. SA was given at either the L3/L4 or L4/L5 level using either 27 G or 25 G of Pencan^®^ or Spinocan^®^ spinal needle. After SA, all parturients were positioned in the supine position with left lateral tilt. Oxygen was supplemented at a rate of 3 L/min via nasal prong. The level of block was determined by pinprick at 5 min and 10 min after spinal administration.

The contents of Syringe A were started at an initial rate of 30 mL/h immediately after the SA. Then, the infusion rate was adjusted according to a treatment algorithm based on NIBP readings. The algorithm aimed to keep maternal SBP within 20% of baseline and above 90 mmHg. According to the algorithm, hypotension was treated by doubling the infusion rate of Syringe A from 30 mL/h to 60 mL/h and administering a 1 mL bolus from Syringe B. In other words, hypotensive patients in Group A received a PE infusion of 100 μg/min, up from 50 μg/min, and hypotensive patients in Group B were given a 100-μg PE bolus. Both patients and anaesthesia team involved were blinded throughout the trial.

All patients underwent a Pfannenstiel incision for CS. SBP, DBP, MAP and HR were recorded every 2 min for 30 min after the delivery of the baby. The infusion protocol was continued until the baby was born. The primary collected data were the series of haemodynamic parameters. The secondary data were the total requirement of PE and Apgar score at 1 min and 5 min after birth.

The sample size was calculated using PS software (Dupont and Plummer 1990). With reference to the study of Choudhary and Bajaj (7), the response within each subject group was normally distributed with a standard deviation of 12.36. Under the assumption that the actual difference between the experimental and control means is 8.58, for the power of study to be 0.8, 34 experimental subjects and 34 control subjects were required to reject the null hypothesis. The type I error probability associated with this test of the null hypothesis was 0.05.

All data were analysed using Statistical Package for Social Science (SPSS) version 26.0. The independent *t*-test or Mann-Whitney U test was used to compare numerical data, and the Pearson’s chi-square or Fisher’s exact test was used to compare categorical data between the two groups. Two-way repeated measures ANOVA was used for the serial haemodynamic parameters. *P*-value < 0.05 was considered significant.

## Results

A total of 74 parturients were randomised into Group A (PE infusion) and Group B (PE bolus). All subjects received the allocated intervention. However, six parturients had incomplete data; thus, only 68 parturients were included for analysis. There were no significant differences in the baseline characteristics of parturients between these two groups, as shown in Table 1.

**Figure 1:**
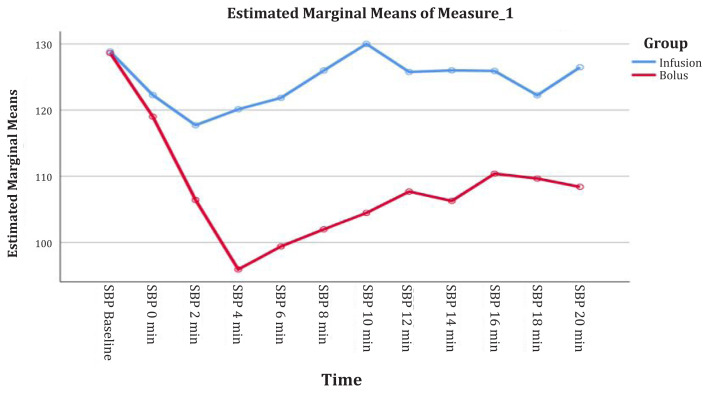
The mean of SBP versus time

The two-way repeated measures ANOVA was used to identify the differences in the means of all parameters. Significantly lower mean SBP was observed in in Group B than in Group A (*F* = 9.31, *P* = 0.00), regardless of time. The overall mean difference between groups was 16.3 (2.1) (Figure 1, Table 2).

**Figure 2:**
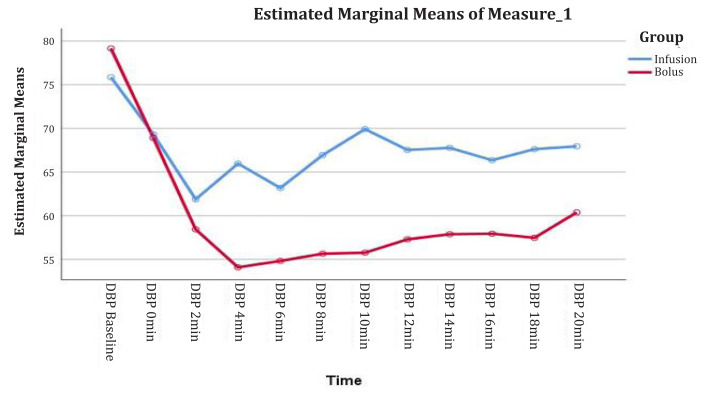
The mean of DBP versus time

The mean DBP was significantly lower in Group B across all times (*F* = 20.7 with *P*-value = 0.000 < α = 0.05). The overall mean difference between the two groups was 7.7 (2.0) mmHg (Figure 2).

**Figure 3:**
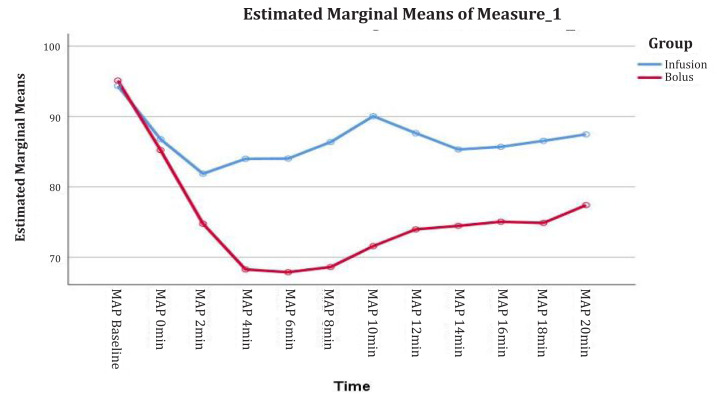
The mean of MAP versus time

The overall mean MAP was significantly lower in Group B than in Group A (*F* = 19.56, *P*-value = 0.00 < α = 0.05). The mean difference between the two groups was 11.1 (1.9) (Figure 3).

The mean total PE requirement was significantly higher in Group A than in Group B at 817.7 mcg (265.7) versus 360.6 mcg (156.0) (*P*-value = 0.0) (Table 3).

All patients had an Apgar score of 8 at 1 min and 9 at 5 min (Table 3).

## Discussion

In obese women, more severe hypotension occurs following SA because of the sympathetic vasomotor block. Although the current evidence supports the use of PE infusion to prevent post-SA hypotension, most studies in the literature regarding PE infusion did not target the obese population.

This study was conducted on obese parturients with BMI between 30 kg/m^2^ and 40 kg/m^2^ and showed significant findings of more stable haemodynamics in the group that received prophylactic PE infusion. The maximum mean percentage reduction of SBP in the infusion group was only 8.57 ± 13.2 mmHg%, whereas the fall in the bolus group was 25.14 ± 11.9 mmHg%. These findings align with a previous study done by Choudhary and Bajaj (7) on PE bolus versus infusion for maternal hypotension and neonatal outcomes. The mean fall in SBP was −28.06 ± 5.3 mmHg% in the bolus group and only −0.44 ± 4.3 mmHg% in the infusion group. However, this study did not include parturients with BMI higher than 30kg/m2.

Our study also revealed that the mean DBP and MAP values were significantly different between the two groups. The reduction of both DBP and MAP was lower in the infusion group than in the bolus group. The results are consistent with the findings demonstrated by Shafeinia et al.’s (8) study on non-obese parturients who underwent CS. The study showed that subjects in the intervention group who had received a PE infusion at 35 mcg/min experienced lesser reductions in MAP, SBP and DBP than the control group.

Prolonged hypotension can impair uterine blood flow and lead to foetal acidosis. Sustained spinal hypotension has been identified as an independent risk for neonatal acidosis, whereas the risk for neonatal acidosis did not increase in women who experienced sporadic post-spinal hypotension only. Thus, preventive strategies for post-spinal hypotension are better than the treatment of established hypotension in others to avoid prolonged hypotension and improve neonatal outcomes (9–12).

In our study, the prophylactic infusion rate began at 50 mcg/min and was adjusted according to NIBP. The starting infusion rate was chosen based on previous studies and recommendations (6, 10). The study revealed that 25 mcg/min and 50 mcg/min fixed-rate PE infusions provided greater maternal BP stability than PE infusions at 75 mcg/min and 100 mcg/min. These findings were consistent with the AAGBI consensus statement published in 2018 by Kinsella et al. (6). The infusion algorithm aims to maintain BP within 20% of baseline and above 90 mmHg, as suggested by AAGBI.

The requirement of PE to maintain BP stability in obese parturients might differ from that of the non-obese population as obesity can alter the pharmacodynamic effects of PE dosing when administered as an infusion or bolus. Our study revealed that the prophylactic PE infusion provided more excellent haemodynamic stability but was associated with a higher total dosage of PE required.

There was no adverse neonatal outcome found from our study in the intervention group. All neonates had an Apgar score of 8 at 1 min and 9 at 5 min. This finding is similar to those of other studies.

The current study only recruited normotensive obese parturients undergoing elective CS with BMI between 30 kg/m^2^ and 40 kg/m^2^. More studies are needed to investigate the benefit of PE infusion on parturients with hypertensive disease, with BMI more than 40 kg/m^2^ and who require emergency CS for foetal distress.

## Conclusion

In conclusion, prophylactic PE infusion provides more excellent haemodynamic stability concerning SBP, DBP and MAP in obese parturients with BMI between 30 kg/m^2^ and 40 kg/m^2^. In addition, PE infusion is not associated with adverse neonatal outcomes.

## Figures and Tables

**Table 1:** Patient’s demographic and clinical characteristics

Variable	Group A (PE Infusion) (*n* = 34)	Group B (PE bolus) (*n* = 34)	*P*-value
Age (years old)	32.7 (5.1)	31.6 (5.4)	0.384
Weight (kg)	83.0 (13.5)	84.1 (12.1)	0.735
Height (cm)	157.5 (5.2)	155.1 (3.5)	0.088
BMI (kg/m^2^)	33.7 (3.3)	34.5 (3.5)	0.341

Note: All numerical data are expressed in mean (SD) and categorical data in *n* (%)

**Table 2:** Percentage change in SBP

Percentage change	Group A (PE Infusion) (*n* = 34)	Group B (PE bolus) (*n* = 34)	*t*	df	*P*-value

	mean (SD)	mean (SD)			
SBP 0 min	−4.9 (12.4)	−7.4(8.8)	0.948	66	0.346
SBP 2 min	−8.6 (13.2)	−16.9(10.0)	2.932	66	0.005
SBP 4 min	−6.5 (12.1)	−25.1(11.9)	6.366	66	0.000
SBP 6 min	−4.9 (15.8)	−22.2 (13.6)	4.798	66	0.000
SBP 8 min	−1.9 (11.9)	−20.4 (10.5)	6.778	66	0.000
SBP 10 min	1.1 (14.4)	−18.4 (12.6)	5.954	66	0.000
SBP 12 min	−1.9 (20.8)	−15.8 (10.3)	3.488	48.24	0.001
SBP 14 min	−1.8 (12.9)	−16.7 ± 17.5	4.003	66	0.000
SBP 16 min	−1.8 (13.4)	−13.5 ± 14.5	3.466	66	0.001
SBP 18 min	−5.0 (15.3)	−14.3 ± 10.1	2.961	66	0.004
SBP 20 min	−1.5 (10.4)	−15.3 ± 18.4	3.808	66	0.000
		
Test		**Mauchly’s W**		**Df**	**Sig**.
		
Mauchly’s test of sphericity		0.054		65	0.00
		
Test		***F***		**Df**	**Sig**.
		
Greenhouse-Geisser		9.31		7.41	0.00
		
Pairwise comparisons		**Mean (SD)**		**95% CI** b	***P*** **-value**
		
Infusion-bolus		16.3 (2.1) *		(12.1, 20.4)	0.00

Notes: Based on estimated marginal means;

* The mean difference is significant at the 0.05 level;

b Adjustment for multiple comparison: Bonferroni

**Table 3:** The mean requirement of PE and the Apgar score of neonates

Variable	Group A (PE infusion) (*n* = 34)	Group B (PE bolus) (*n* = 34)	*P*-value	
**Phenylephrine (mcg)**	817.7 (265.7)	360.6 (156.0)	0.00	

	**Group A** **(PE Infusion)** **(** ***n*** ** = 34)**		**Group B** **(PE bolus)** **(** ***n*** ** = 34)**	

	median (IQR)	95% CI	median (IQR)	95% CI
Apgar scores at 1 min	8 (0)	8-8	8 (0)	8-8
Apgar scores at 5 min	9 (0)	9-9	9 (0)	9-9

Notes: All numerical data are expressed in mean (SD) and categorical data in *n* (%)

## References

[1] Weiss JL, Malone FD, Emig D, Ball RH, Nyberg DA, Comstock CH (2004). Obesity, obstetric complications and Caesarean delivery rate—a population-based screening study. Am J Obstet Gynecol.

[2] Klöhr S, Roth R, Hofmann T, Rossaint R, Heesen M (2010). Definitions of hypotension after spinal anaesthesia for caesarean section: Literature search and application to parturients. Acta Anaesthesiol Scand.

[3] Nani FS, Torres ML (2011). Correlation between the body mass index (BMI) of pregnant women and the development of hypotension after spinal anesthesia for Caesarean section. Rev Bras Anestesiol.

[4] Anesthesiologists Task Force on Obstetric Anesthesia and the Society for Obstetric Anesthesia and Practice Guidelines for Obstetric Anesthesia (2016). An updated report by the American Society of Perinatology. Anesthesiology.

[5] Ngan Kee WD, Khaw KS, Ng FF, Lee BB (2004). Prophylactic phenylephrine infusion for preventing hypotension during spinal anesthesia for caesarean delivery. Anesth Analg.

[6] Kinsella SM, Carvalho B, Dyer RA, Fernando R, McDonnell N, Mercier FJ (2018). International consensus statement on the management of hypotension with vasopressors during caesarean section under spinal anaesthesia. Anaesthesia.

[7] Choudhary M, Bajaj JK (2018). Study comparing phenylephrine bolus and infusion for maternal hypotension and neonatal outcome during Caesarean section under spinal anesthesia. Anesth Essays Res.

[8] Shafeinia A, Ghaed MA, Nikoubakht N (2020). The effect of phenylephrine infusion on maternal hemodynamic changes during spinal anesthesia for Caesarean delivery. Anesth Pain Med.

[9] Siddik-Sayyid SM, Taha SK, Kanazi GE, Aouad MT (2014). A randomized controlled trial of variable rate phenylephrine infusion with rescue phenylephrine boluses versus rescue boluses alone on physician interventions during spinal anesthesia for elective caesarean delivery. Anesth Analg.

[10] George RB, McKeen DM, Dominguez JE, Allen TK, Doyle PA, Habib AS (2018). A randomized trial of phenylephrine infusion versus bolus dosing for nausea and vomiting during Caesarean delivery in obese women. Can J Anaesth.

[11] Allen TK, George RB, White WD, Muir HA, Habib AS (2010). A double-blind, placebo-controlled trial of four fixed rate infusion regimens of phenylephrine for hemodynamic support during spinal anesthesia for cesarean delivery. Anesth Analg.

[12] Knigin D, Avidan A, Weiniger CF (2020). The effect of spinal hypotension and anesthesia-to-delivery time interval on neonatal outcomes in planned Caesarean delivery. Am J Obstet Gynecol.

